# Relationship between Physical Activity Level and Depression of Elderly People Living Alone

**DOI:** 10.3390/ijerph16204051

**Published:** 2019-10-22

**Authors:** Haewon Byeon

**Affiliations:** Department of Speech Language Pathology, School of Public Health, Honam University, 417, Eodeung-daero, Gwangsan-gu, Gwangju 62399, Korea; bhwpuma@naver.com; Tel.: +82-10-7404-6969

**Keywords:** physical activity, depression, elderly people living alone, Patient Health Questionnaire-9, flexibility exercise, muscular strength exercise, complex sample logistic regression

## Abstract

Background and objectives: Only a few studies analyzed the physical activity level of elderly people living alone in local communities and evaluated the relationship between it and mental health. The purpose of this study was to investigate the relationship between regular physical activity and depression in the elderly living alone and to provide basic data for the prevention of depression in the elderly. Materials and Methods: We analyzed 256 elderly people living alone aged 65 years or older who completed the 2014 Korea National Health and Nutrition Examination Survey. Depression was defined as a score of 10 or higher using Patient Health Questionnaire-9 (PHQ-9). This study investigated walking per week, days of muscular strength exercise performance in the past 1 week, days of flexibility exercise in the past 1 week, mean hours in a sitting position per day, the numbers of days and hours conducting a high intensity physical activity in the past 1 week, and numbers of days and hours conducting a medium intensity physical activity in the past 1 week to define physical activity. Our study presented prevalence odds ratios (pOR) and 95% confidence interval (CI) by using complex sample logistic regression analysis in order to identify the relationship between physical activity and depression. Results: The results of complex sample logistic regression analysis showed that flexibility exercise was significantly related to depression (p < 0.05). On the other hand, the mean hours in a sitting position per day, aerobic physical activity, walking, and muscular strength exercise were not significantly related to geriatric depression. Conclusions: The results of our study implied that persistent flexibility exercise might be more effective to maintain a healthy mental status than muscular strength exercise. A longitudinal study is required to prove the causal relationship between physical activity and depression in the old age.

## 1. Introduction

As the world is experiencing aging, geriatric depression has become an important health issue. It has been known that depression triggers enormous socioeconomic costs by causing suicidal thoughts, lower work efficiency, and increased medical costs [1]. The five major causes of death and disability in the world were pneumonia, diarrhea, fall, depression, and ischemic heart diseases, in the order of magnitude, in 1990 [1]. However, depression is projected to be second after ischemic heart diseases in 2020 [1]. The world’s economic burden due to these non-infectious diseases from 2011 to 2030 is almost $47 trillion, including direct treatment cost and reduced productivity owing to the pain of patients and their caregivers [2]. Particularly, the economic burden due to mental illness is $16.3 trillion, accounting for the major portion of the overall burden [2]. The socioeconomic costs of depression have steadily increased over the past decade in South Korea as well [3]. As of 2013, the socioeconomic costs of depression are estimated as $8.9 billion [3]. Since it is projected that the elderly population (≥60 years old) will be doubled by 2050, the occurrence rate of depression and associated costs will also increase inevitably. 

Particularly, South Korea is experiencing aging at the fastest pace in the world. Since South Korea is expected to enter into a super-aged society, more attention is needed to diagnose, intervene, and prevent geriatric depression as soon as possible. The Ministry of Health and Welfare (2014) [4] conducted a study using “Short Form of Geriatric Depression Scale” in South Korea and reported that 33.1% of the elderly experienced a depressive symptom. Lee (2008) [5] also showed that 35–60% of the elderly in South Korea expressed a mild or worse depressive symptom. Depression is defined as a syndrome in that one experiences a depressed mood for at least 2 weeks or becomes not interested in or loses pleasure in most activities while the emotional issue is not caused by a temporary medicine (such as steroids) or a medical condition (such as delirium) [5]. A person with depression experiences demotivation and lethargy. Particularly, geriatric depression is known as a critical factor to diminish medical treatment effects and increase suicidal ideation [6,7,8]. Although depression can be fully recovered, geriatric depression, when it is not treated properly, is likely to cause not only interpersonal problems but also the deterioration of cognitive functions such as the deterioration of memory, concentration, and computation ability [9]. Therefore, identifying the risk factors and prevention factors is an important social issue. 

On the other hand, demographic data show that the number of elderly people who live alone is rapidly increasing in South Korea. Statistics Korea (2018) [10] reported that the number of elderly people living alone increased from 115,000 people in 1985 to 1,340,000 people in 2017, more than a 10-fold increase. It was also reported that the proportion of those living alone in the entire elderly households increased ten times from 8.9% in 1990 to 19.3% in 2017. It is projected that the proportion of those living alone will increase due to weakened parental caregiving consciousness and the new lifestyle of healthy and wealthy elderly people who prefer independent living. Therefore, it is urgent to study the mental health of elderly people living alone. 

Many previous studies have reported that active physical activities can prevent geriatric depression [11]. Physical activity is defined as the basic activity of all human beings that consume energy through the contraction and relaxation of musculi skeleti, and is a broad concept encompassing all body movements in everyday life [12]. It has been reported that continuous physical activity would decrease depression [13]. The elderly who performed a physical activity (e.g., aerobic exercise, muscular strength exercise, muscular endurance exercise, and combined exercise) regularly experienced depression significantly less than those who did not exercise [13]. Kim (2015) evaluated factors affecting the occurrence of depression using 329 elderly people who were living in local communities of South Korea and showed that the elderly who conducted a moderate intensity physical activity (e.g., power walking, jogging, and dance sports) for 150 minutes or more per week had significantly less (54% less) risk of suffering depression than those who did not perform a moderate intensity physical activity [14]. Moreover, it was also reported that the elderly who were not engaged in a physical activity had a higher chance of developing depression [11]. 

Although the number of the elderly living alone in South Korea is increasing due to demographic changes, the topic of previous studies mainly focused on a few topics: (1) some studies [14] did not adjust household composition (e.g., elderly living alone) or evaluated subjects living in one area; (2) some studies [14] considered the elderly (≥65 years old) as one group although the number of the advanced elderly (≥75 years old) was increasing, and examined the prevalence of depression; and (3) other studies [15] emphasized that the mental health of elderly people living alone was poorer than those living with a spouse or a child. Older people may have different physical characteristics according to young-old (ages 65–74) and the middle-old (ages 75–84) [14]. Also, only a few studies have evaluated associative factors (e.g., positive relationship and negative relationship) in depression in elderly people living alone. Since various factors such as socioeconomic level and health status can influence physical activity levels [16], it is difficult to assume that physical activity characteristics would be related to the depression of elderly people living alone even though the relationship was proven for the elderly living with another family member. The purpose of this study was to investigate the relationship between physical activity and depression in the elderly living alone and to provide basic data for the prevention of depression in the elderly.

## 2. Materials and Methods

This is a secondary data analysis study using the raw data of the Korea National Health and Nutrition Examination Survey (KNHANES) conducted from 1 January to 31 December 2014. The KNHANES is an epidemiological survey conducted by the Korea Center for Disease Control and Prevention with the support of the Ministry of Health and Welfare in order to generate statistics that can present the health level and health behaviors of South Korean population reliably and representatively based on the National Health Promotion Act (Article 16). The questionnaire items of the KNHANES (ex. Demographic variables, physical activity and health behaviors) are based on the National Health and Nutrition Examination Survey in the United States. Please refer to Korea Centers for Disease Control and Prevention [17] for further details of these survey items. The survey was approved by the IRB of the Korean Center for Disease Control and Prevention (2013-12EXP-03-5C). 

The KNHANES was conducted on samples extracted by the two-stage stratified cluster sampling method and is representative of South Koreans living in local communities. The sample excluded South Koreans who were hospitalized or in correctional facilities at the time of sampling. The 2014 survey had 7500 subjects from 4600 households in 200 areas across South Korea. The values obtained from the KNHANES were multiplied with independently estimated weights in order to make the samples represent the overall Korean population. The sample weight of KNHANES was created, the base weight was calculated, adjustments for non-response were made, and post-stratification adjustments were made using the 2005 South Korea Census of Population and Housing. Please refer to Korea Centers for Disease Control and Prevention [17] for further details of these weights. 

This study first selected 327 elderly people living alone from 1586 elderly people (≥65 years old), who completed both a health survey and depression screening test of the KNHANES, by excluding 1259 elderly people living with a family member. Afterward, this study eliminated 71 subjects who did not respond to Patient Health Questionnaire-9 (PHQ-9) [18], a depression screening test, from the 327 subjects. The final analysis subjects of this study were 256 people and, when the weights are reflected, the samples represent 971,709 elderly people living alone in South Korea. 

### 2.1. Measurement

#### 2.1.1. Depression

Depression was defined using PHQ-9 referring to An et al. (2013) [19]. PHQ-9 is a self-reporting type depression screening tool that was standardized according to the major depression diagnostic criteria of the Diagnostic and Statistical Manual of Mental Disorders (4^th^ edition; DSM-IV) [20]. PHQ-9 consists of nine items corresponding to the depression diagnostic criteria of the DSM-IV and examines how often a subject has experienced these problems in the past 2 weeks. The response is rated on a 4-point scale (never = 0 point; 3–4 days = 1 point; 8–10 days = 2 points; and 12–14 days = 3 points). The range of scores is between 0 and 27 points, and depression is defined as 10 points and higher (Han et al. 2008) [18]. Han et al. (2008) reported that PHQ-9 had higher sensitivity and specificity than other depression screening tests such as General Health Questionnaire-12 or World Health Organization Five-Item Well-Being Index. An et al. (2013) [19] conducted a study targeting the local population and showed that the sensitivity and specificity of this test were 0.89 and 0.95, respectively. Moreover, the Cronbach’s alpha of this study was 0.86.

#### 2.1.2. Physical Activity 

In this study, regular physical activities performed on average in a week were investigated using a self-care questionnaire. This study investigated days of walking (at least 10 minutes per time; e.g., commuting, trip, and exercise) per week (no, 1–3 days, 4–6 days, or 7 days), days of muscular strength exercise performance (e.g., push-up, sit-up, dumbbell, barbell, or pull-up bar at least 10 minutes per time) in the past 1 week (no, 1–4 days, or 5 days or more), days of flexibility exercise (e.g., stretching and freehand exercise at least 10 minutes per time) in the past 1 week (no, 1–4 days, or 5 days or more), mean hours in a sitting position per day (4 hours or less, 5–8 hours, or 9 hours or more), the numbers of days and hours conducting a high-intensity physical activity (e.g., running, jump rope, hiking, playing basketball, swimming, and playing badminton) in the past 1 week, and numbers of days and hours conducting a medium-intensity physical activity (e.g., power walking, jogging, weight training, playing golf, and playing dance sports) in the past 1 week to define physical activity. This study measured aerobic physical activity (yes or no), where ‘yes’ means one conducted a medium intensity physical activity for 150 minutes or more or a high-intensity physical activity for 75 minutes or more, using the data of the medium intensity and high-intensity physical activity in the past 1 week. 

#### 2.1.3. Covariate 

Covariates included gender, age (Young-Old): 65–74 years old; Old-Old: 75 years old or more), education level (elementary school graduate or less, or middle school graduate or more), residential area (urban or rural), household income quartile, economic activity (e.g., work life after retirement age; yes or no), private health insurance subscription (yes or no), smoking (non-smoker, past smoker, or current smoker), the mean frequency of binge per week (no, one or less per week, or two or more per week), and pain and discomfort (yes or no) due to an illness or an accident during the past 2 weeks. This study defined binge as the alcohol consumption of 61 g or more per drinking (seven shots of soju or more) for males and that of 42 g or more per drinking (five shots of soju or more) for females based on the criteria of the International Center for Alcohol Policies (2005) [21].

#### 2.1.4. Statistical Analysis

This study used SURVEY procedure, which applies weights in order to make the samples be representative of the elderly population in South Korea, considering that the KNHANES collected data using a stratified, clustered, and systematic sampling [17]. The weighted percentages and standard errors for the general characteristics of the subjects were presented using frequency analysis after applying weights. The differences in the characteristics between groups were analyzed by using weight applied Rao-Scott chi-square test. Moreover, this study presented prevalence odds ratios (pOR) and 95% confidence interval (CI) by using complex sample logistic regression analysis in order to identify the relationship between physical activity and depression. The study model was composed of model 1, model 2, and model 3. The model 1 adjusted demographic factors (i.e., gender, age, residential area, educational level, household income, economic activity, and subscribed health insurance type). Model 2 additionally adjusted health behaviors (i.e., drinking and binge) from the Model 1. Model 3 additionally adjusted health factors (i.e., pain and discomfort in the past 2 weeks) from model 2. All analyses were conducted using Stata 13.1^®^ (StatCorp, Houston, Texas, USA), and statistical significance was determined at alpha = 0.05 in a two-sided test.

## 3. Results

### 3.1. General Characteristics of Subjects 

The general characteristics of the subjects are shown in Table 1. Young-Old (65–74 years old) accounted for 49.4%, and Old-Old (75 years and older) was 50.6%. The majority of the samples were females (83.9%). The results showed that 79% of subjects were elementary school graduate or below, 72.7% were economically inactive people, 79.2% were in the first income quartile, 73.6% lived in an urban area, 78.2% were non-smokers, 89.3% did not go on a binge, 63.2% did an aerobic physical activity, 45% spent more than 9 hours in a sitting position, and 90% did not do a muscular strength exercise. The results of PHQ-9 test indicated that 19% of the subjects had depression.

### 3.2. Characteristics of Subjects by the Prevalence of Depression

Table 2 shows the general characteristics of subjects by the prevalence of depression. The results of Rao-Scott chi-square test revealed that the prevalence of depression was significantly (p < 0.05) affected by gender (p < 0.001), education level (p = 0.003), economic activity (p = 0.007), smoking (p = 0.006), pain and discomfort in the past 2 weeks (p = 0.002), and the days of flexibility exercise per week (p = 0.013). The prevalence of depression was high when subjects were females, elementary school graduation and below, economically inactive, and non-smokers, had pain or discomfort in the past 2 weeks, and did not do flexibility exercise.

### 3.3. Relationship between a Physical Activity and Depression 

The results of complex sample logistic regression analysis showed that flexibility exercise was significantly (p < 0.05) related to depression (Table 3). Even after adjusting all compounding variables (model 3), the elderly who conducted flexibility exercise between 1 and 4 days had an approximately 81% lower risk to suffer from depression than those who did not do it (pOR = 0.19, 95% CI: 0.05–0.75), and those who conducted it 5 days or more had an approximately 66% lower risk to suffer from depression than those who did not do it (pOR = 0.34, 95% CI: 0.11–0.99). On the other hand, the mean hours in a sitting position per day, aerobic physical activity, walking, and muscular strength exercise were not significantly related to geriatric depression.

## 4. Discussion 

This is a cross-sectional study, and the results of this study showed that regular flexibility exercises were independently related to depression prevention. Many studies on the relationship between the physical activity of the elderly and depression in local communities also reported that physical activity programs significantly decreased depression [11]. The physical activity of the elderly in local communities is a way to enhance the physical and mental health by increasing the physical fitness and intellectual activity in everyday life and is known to be more effective than other lifestyle habits [22]. Blumenthal et al. (1999) [23] analyzed the effects of physical activities on the elderly with a major depressive disorder, and reported that an aerobic exercise program (three times per week for 16 weeks) was effective in treating it and that the effects were statistically equivalent to the treatment effects of sertraline hydrochloride, an antidepressant. According to the Consensus Statement [24] published by the National Institute of Mental Health after comprehensively examining previous studies, a physical activity has positive effects on mental health and the psychological well-being of an individual. Particularly, it reported that regular exercise decreased anxiety, depression, and stress gradually, regardless of age and gender. 

How the physical activity of the elderly decreases depression may be explained in two ways. The first is that the elderly who are continuously engaged in physical activity experience positive leisure activities and social support more, and it results in higher psychological well-being and ultimately reduces depression. Continuous physical activity strengthens social support such as enhancing friendship, exchanging emotional aids, and discussing person issues [25]. Physical activity can ultimately decrease depression in everyday life and positively affect mental health through it. Fox (1999) [26] examined the relationship between social support and psychological well-being through physical activities and reported that continuous participation of the elderly in a physical activity decreased depression and that these participants experienced higher social support and subjective happiness than non-participants. Lee et al. (2016) [27] also indicated that regular exercise could decrease geriatric anxiety and depression regardless of gender. It will be needed to test the relationship between physical activity and geriatric depression in various races and cultures in the future.

The second is that participation in a physical activity could increase the physical strength of a participant and give a positive mood to a participant to improve the ability of the participant to cope with depression. Physical activities are known to increase the secretion of antidepressant hormones: Physical activities can change the central norepinephrine activity temporarily, decrease the hypothalamopituitary–adrenocortical axis, and increase the secretion of beta-endorphins [28]. Therefore, continuing physical activity can maintain a positive mood, and ultimately prevent depression that is manifested by a negative mood [29]. Particularly, depression experienced by elderly people living alone (e.g., caused by bereavement) is generally chronic, rather than acute, and it decreases the satisfaction of life [30]. The results of this study imply that continuous physical activity can have a positive impact on reducing the depression of the elderly. 

Another meaningful finding of this study was that flexibility exercise such as stretching, freehand exercise, and yoga was significantly related to the prevention of depression. It is also known that flexibility exercise may enhance the mental health of the elderly. For example, Wang et al. (2014) [31] showed that tai chi significantly decreased depression. Rogers et al. (2009) [32] also concluded after reviewing 36 studies that tai chi not only enhanced the physical function of the elderly but also effectively decreased the depression and anxiety of them. Moreover, it was confirmed that yoga had grade B evidence in reducing depression [33]. Nevertheless, South Korea still does not have enough programs providing flexibility physical activities for the elderly living alone [33], and there are not enough studies on the relationship between their psychological variables and their physical activity level and type. More intervention studies and epidemiological studies are needed to scientifically demonstrate the impact of physical activities on mental health in the old age. 

The limitations of this study are as follows. First, this study only analyzed the prevalence of depression using a depression screening test, so the severity of depression was not identified. Future studies are required to identify the severity of depression and to demonstrate its relationship to physical activity. Secondly, although there are close relationships among physical activities, participation in a social activity (e.g., the frequency of contacting neighbors) and depression in the old age, this study did not investigate the participation in a social activity and the social activity type of elderly people living alone. Future studies are needed to evaluate the relationship between a physical activity and depression by adding participation in a social activity in a study model. Thirdly, this study examined average physical activity over the past week. However, the cumulative period of physical activity has not been investigated. If the physical activity is repeated regularly, after a few weeks, changes occur known as training-induced adaptations, which persist for longer than the temporary acute changes. Future research will need to identify dose-response effects. Fourth, in this epidemiologic study, we did not include the presence of chronic diseases (ex. The number of chronic diseases) in the confounding variables. Chronic conditions are related to depression which may act as a mediator to moderate-to-vigorous physical activity. Future research will need to include chronic disease as a confounding variable. Fifth, even if this study proves a relationship between physical activity and depression, the result cannot be interpreted as a causal relationship because it is a cross-sectional study. Therefore, the results should be interpreted carefully. 

## 5. Conclusions

The flexibility exercise of the elderly was independently associated with depression prevention. The results of this study implied that persistent flexibility exercise (e.g., stretching and freehand exercise) might be more effective to maintain a healthy mental status than muscular strength exercise. A longitudinal study is required to prove the causal relationship between physical activity and depression in old age.

## Figures and Tables

**Table 1:** Sample characteristics (n = 256).

Characteristics	n (Weighted n)	Weighted %
Gender		
Male	46 (156,551)	16.1
Female	210 (815,158)	83.9
Gender		
Male	135 (480,179)	49.4
Female	121 (291,530)	50.6
Education level		
Elementary school graduate or less	200 (767,365)	79.0
Middle school graduate or more	56 (204,344)	21.0
Residential area		
Urban	181 (714,743)	73.6
Rural	75 (256,967)	26.4
Household income quartile		
Q1	200 (766,015)	79.2
Q2	40 (158,230)	16.4
Q3	11 (34,074)	3.5
Q4	3 (8520)	0.9
Economic activity		
Yes	74 (265,417)	27.3
No	182 (706,292)	72.7
Private health insurance subscription		
Yes	63 (235,789)	24.3
No	191 (729,312)	75.1
Smoking		
Non-smoker	189 (715,422)	78.2
Past smoker	29 (104,197)	11.4
Current smoker	25 (95,599)	10.4
The mean frequency of binge per week		
No	218 (823,997)	89.3
One or less per week	25 (90,596)	9.8
Two or more per week	2 (8386)	0.9
Pain and discomfort due to an illness or an accident during the past two weeks		
Yes	111 (424,561)	43.7
No	145 (547,148)	56.3
Aerobic physical activity		
Yes	159 (611,117)	63.2
No	95 (355,086)	36.8
Mean hours in a sitting position per day		
4 hours or less	44 (181,569)	20.0
5–8 hours	81 (316,839)	35.0
9 hours or more	113 (407,814)	45.0
Walking per week		
No	79 (309,033)	32.1
1–3 days	52 (186,161)	19.3
4–6 days	50 (201,405)	20.9
7 days	73 (265,629)	27.6
Days of flexibility exercise in the past one week		
No	158 (602,883)	62.0
1–4 days	47 (179,790)	18.5
5 days or more	51 (189,036)	19.5
Days of muscular strength exercise performance in the past one week		
No	230 (868,663)	90.0
1–4 days	16 (65,692)	6.8
5 days or more	9 (30,561)	3.2
Depression		
Yes	45 (184,691)	19.0
No	211 (787,018)	81.0

**Table 2:** Characteristics of subjects by the prevalence of depression, n (weight %).

Characteristics	Depression (n = 256)	
	No (n = 211)	Yes (n = 45)
Gender		
Male	46 (100)	0 (0)
Female	165 (77.3)	45 (22.7)
Age		
65–74	112 (82.0)	23 (18.0)
75+	99 (80.0)	22 (20.0)
Education level		
Elementary school graduate or less	158 (77.3)	42 (22.7)
Middle school graduate or more	53 (94.9)	3 (5.1)
Residential area		
Urban	148 (79.8)	33 (20.2)
Rural	63 (84.4)	12 (15.6)
Household income quartile		
Q1	159 (78.2)	41 (21.8)
Q2	36 (88.7)	4 (11.3)
Q3	11 (100)	0 (0)
Q4	3 (100)	0 (0)
Economic activity		
Yes	68 (92.4)	6 (7.6)
No	143 (76.7)	39 (23.3)
Private health insurance subscription		
Yes	54 (85.7)	9 (14.3)
No	155 (79.3)	36 (20.7)
Smoking		
Non-smoker	149 (77.4)	40 (22.6)
Past smoker	28 (95.6)	1 (4.4)
Current smoker	25 (100)	0 (0)
The mean frequency of binge per week		
No	177 (80.3)	41 (19.7)
One or less per week	24 (92.2)	1 (7.8)
Two or more per week	2 (100)	0 (0)
Pain and discomfort due to an illness or an accident during the past 2 weeks		
Yes	82 (71.5)	29 (28.5)
No	129 (88.3)	16 (11.7)
Aerobic physical activity		
Yes	128 (78.1)	31 (21.9)
No	81 (85.7)	14 (14.3)
Mean hours in a sitting position per day		
4 hours or less	37 (80.8)	7 (19.2)
5–8 hours	72 (88.8)	9 (11.2)
9 hours or more	87 (74.6)	26 (25.4)
Walking per week		
No	60 (74.8)	19 (25.2)
1–3 days	45 (83.5)	7 (16.5)
4–6 days	42 (83.5)	8 (16.5)
7 days	62 (83.9)	11 (16.1)
Days of flexibility exercise in the past 1 week		
No	122 (75.4)	36 (24.6)
1–4 days	44 (93.7)	3 (6.3)
5 days or more	45 (86.8)	6 (13.2)
Days of muscular strength exercise performance in the past 1 week		
No	187 (80.0)	43 (20.0)
1–4 days	16 (100)	0 (0)
5 days or more	8 (86.6)	1 (13.4)

**Table 3:** Relationship between a physical activity and depression: complex sample logistic regression model, pOR (95% CI).

Variable	Model 1	Model 2	Model 3
Mean hours in a sitting position per day			
4 hours or less	1	1	1
5–8 hours	0.65 (0.21, 2.02)	1.06 (0.30, 3.75)	0.97 (0.27, 3.46)
9 hours or more	1.22 (0.45, 3.33)	1.95 (0.62, 6.10)	1.62 (0.51, 5.16)
Aerobic physical activity			
No	1	1	1
Yes	0.89 (0.43, 1.86)	0.79 (0.36, 1.74)	0.82 (0.36, 1.84)
Walking per week			
No	1	1	1
1–3 days	0.56 (0.20, 1.53)	0.42 (0.14, 1.28)	0.49 (0.16, 1.52)
4–6 days	0.46 (0.17, 1.26)	0.34 (0.11, 1.03)	0.40 (0.13, 1.26)
7 days	0.70 (0.28, 1.78)	0.56 (0.20, 1.56)	0.76 (0.25, 2.28)
Days of flexibility exercise in the past 1 week			
No	1	1	1
1–4 days	0.21 (0.06, 0.74) *	0.19 (0.05, 0.74) *	0.19 (0.05, 0.75) *
5 days or more	0.40 (0.15, 1.08)	0.31 (0.10, 0.95) *	0.34 (0.11, 0.99) *
Days of muscular strength exercise performance in the past 1 week			
No	1	1	1
1–4 days	NA	NA	NA
5 days or more	0.75 (0.08, 7.36)	0.70 (0.07, 7.09)	0.85 (0.08, 9.06)

* p < 0.05; NA = not analyzed; Model 1 = adjusted with gender, age, residential area, education level, household income, economic activity, and private health insurance subscription; Model 2 = additionally adjusted the model 1 with smoking and binge; and model 3 = additionally adjusted the model 2 with pain and discomfort in the past 2 weeks.
